# Sources and Sinks of Diversification and Conservation Priorities for the Mexican Tropical Dry Forest

**DOI:** 10.1371/journal.pone.0003436

**Published:** 2008-10-17

**Authors:** Judith X. Becerra, D. Lawrence Venable

**Affiliations:** 1 Department of Biosphere 2, University of Arizona, Tucson, Arizona, United States of America; 2 Department of Ecology and Evolutionary Biology, University of Arizona, Tucson, Arizona, United States of America; University of Sheffield, United Kingdom

## Abstract

Elucidating the geographical history of diversification is critical for inferring where future diversification may occur and thus could be a valuable aid in determining conservation priorities. However, it has been difficult to recognize areas with a higher likelihood of promoting diversification. We reconstructed centres of origin of lineages and identified areas in the Mexican tropical dry forest that have been important centres of diversification (sources) and areas where species are maintained but where diversification is less likely to occur (diversity sinks). We used a molecular phylogeny of the genus *Bursera*, a dominant member of the forest, along with information on current species distributions. Results indicate that vast areas of the forest have historically functioned as diversity sinks, generating few or no extant *Bursera* lineages. Only a few areas have functioned as major engines of diversification. Long-term preservation of biodiversity may be promoted by incorporation of such knowledge in decision-making.

## Introduction

The diversity crisis has exacerbated the need for information to direct conservation efforts [1]. Most of the attention has been directed to biodiversity hotspots [2], in particular to areas that contain high species richness or areas with high levels of endemism [3]. The rationale behind conserving hotspots is that protecting these areas should prevent the extinction of a larger number of species than would protecting areas of same size somewhere else. Other targets for conservation have also been proposed, but the common denominator of them all is a focus on current diversity. A problem with this approach is that geographic centres of extant diversity may not coincide with geographic centres of origin, thus conservation of currently defined hotspots may not protect the process of diversification [4].

It has been suggested that conservation policies should also target the maintenance of future diversification [5]. However, even in the best of times it is difficult to predict long-term evolutionary processes. Today the need for immediate action to mitigate the biodiversity crisis exacerbates the problem. Although we only have a rudimentary understanding of how we are altering future evolutionary processes, we may still be able to make meaningful predictions about future diversification based on historical records. It has been recognized that different areas have contributed unevenly to diversification [6]. Some areas have had a higher tendency to function as engines or sources of diversification whiles other tend to maintain species without generating them [5].

Elucidating the geographical history of diversification is critical for inferring where future diversification may occur and thus would be a valuable aid in determining conservation priorities [7]. If not drastically altered by people, areas that historically favoured diversification may be more likely to produce more species in the future. This approach is different from determining diversity hot/cold spots which focuses on finding areas of high extant diversity. Identifying spatial sources and sinks in diversification and combining this information with extant diversity may have greater long-term conservation payoff than considering extant diversity alone [8].

In this paper we have reconstructed diversification sources and sinks and compared them to extant diversity and extant endemism using the speciose genus of tropical trees, *Bursera*. Based on these reconstructions we make predictions about which areas of the Mexican tropical dry forest may be more beneficial to conserve the future diversification of this genus.

The tropical dry forest is one of the four most extensive types of vegetation of Mexico [9]. In its natural state, it is a dense community dominated by low to medium sized trees that lose their leaves during the dry season. This forest is widespread on the Pacific slopes of Mexico covering great extensions from central Sonora and southeastern Chihuahua to the southern state of Chiapas and continuing on to Central America. Although the tropical dry forest contains a high diversity of plants, two groups dominate the woody elements: legumes and the genus *Bursera* (Burseraceae) [10], [11]. *Bursera* comprises ∼100 species of trees distributed from Southern U.S. to Peru [10], [12]. It reaches its maximum diversity and abundance in the tropical dry forests of Mexico where, with about 84 species - 80 of them endemic, it is the most speciose genus and often one of the most abundant groups [9], [11], [13]. The dominance of *Bursera* is especially high along the deep canyons of the Balsas River basin and its tributaries [14]. On the floors and slopes of these canyons this genus is often the absolute dominant woody taxon, surpassing legumes and other groups in diversity and abundance. In the south of Mexico it is common to find 5–15 *Bursera* species coexisting in single localities. The identity of the species often changes from one place to another because of the high level of endemism in the genus. About 65% of the species have a geographic distribution of <50,000 Km^2^. The genus is well adapted to the warm and dry conditions of the dry forest. All of its species are deciduous and most of them cold-sensitive. Because the genus is old, highly adapted to the dry forest, and of great physiognomic importance in this biome today, it has been suggested that its evolution and diversification could be tied to the history of the Mexican tropical dry forests [15]. *Bursera* is also a conspicuous constituent of habitats like desertscrub and thornscrub of the Mexican central and northern deserts, the low-land tropical rain forests along the Pacific and Atlantic coasts and higher altitude woodland forests [9], [11].

Diversification studies have suggested that, although *Bursera* is old, its peak in diversification occurred about 17-10 million years ago. This coincided with the formation of the Western Sierra Madre and the Neovolcanic belt, the mountainous systems that are now recognized as critical for the persistence of the Mexican dry forest [15], [16]. Diversification in the genus continues being high. It is estimated that at least 40 species (∼35% of total number) have originated in the last 7 million years.

## Materials and Methods

**Figure 1:**
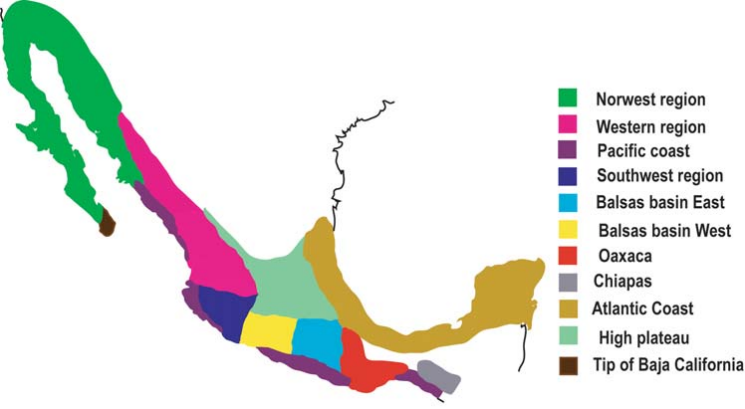
Geography of *Bursera* in Mexico. The distribution of *Bursera* was divided into 11 sub-areas following recognized biogeographic areas for the genus. Modified from Becerra [15].

**Figure 2:**
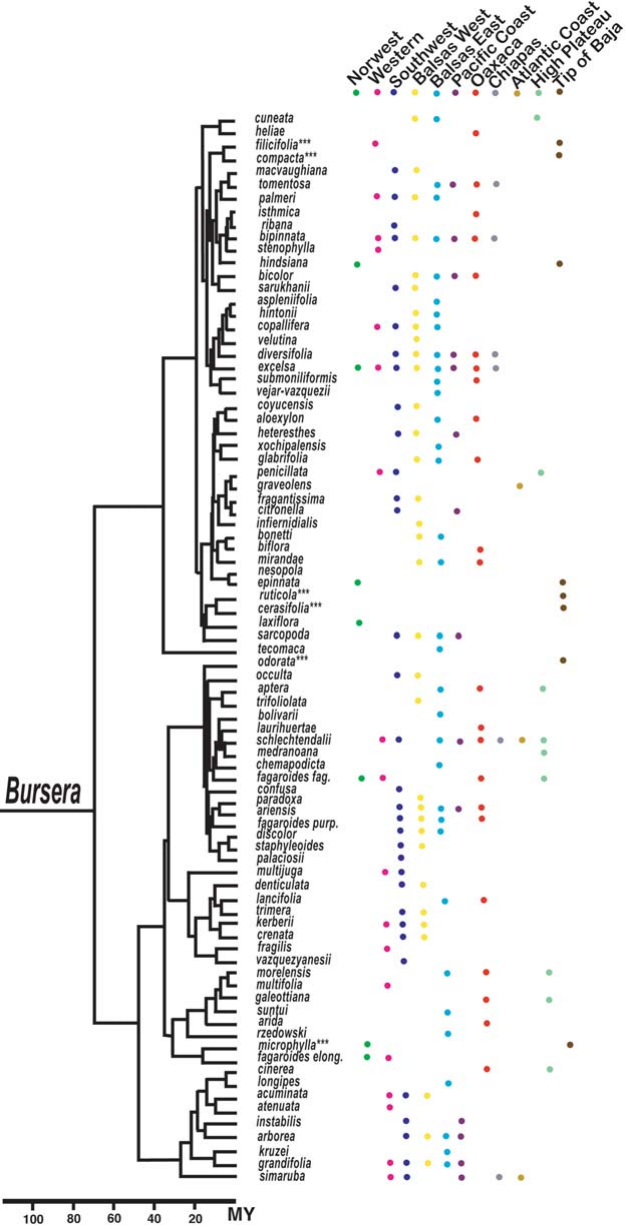
Time-calibrated molecular phylogeny and distribution of species of *Bursera* in the 11 sub-biogeographical areas. Asterisks indicate species that are found in the Cape region of the Baja California peninsula. These species were treated as if their distribution included the south of the state of Jalisco (see materials and methods).

The purpose of our investigation was to identify geographic areas of high diversification as a way to help make predictions about future diversification for the genus *Bursera*. For this, maps of current distribution were generated for each species using information from herbarium specimens [the Universidad Nacional Autonóma de Mexico Herbarium (MEXU), the Escuela Nacional de Ciencias Biológicas, Instituto Politécnico Nacional Herbarium (ENCB), and the herbarium of the Instituto de Ecología, Bajío, México Instituto de Ecología, A.C. (IEB), from the on-line biodiversity information of the Mexican Comisión para el Conocimiento y Uso de la Biodiversidad (CONABIO, www.conabio.gob.mx), and from visits to many sites in the last 16 years. *Bursera*'s current distribution in Mexico was divided into 11 sub-areas according to biogeographic information published on the genus and well-known biogeographic areas for the Mexican vegetation [15], [17], [18] (Figs. 1and 2). The selected areas were: 1) the northwest region, 2) the western region, 3) the sub-humid forests of the Pacific coast, 4) the southwest region, 5) the eastern side of the Balsas basin, 6) the western side of the Balsas basin, 7) the tropical dry forests of Oaxaca (excluding the ones in the eastern side of the Balsas basin), 8) the Chiapas region, 9) the Atlantic coast, 10) the tropical dry forests at the southern tip of Baja California, and 11) the central high plateau.

To reconstruct centres of origin of lineages we used a robust time-calibrated DNA phylogeny recently reconstructed for *Bursera* [10], [15]. This reconstruction used sequences from the internal transcribed spacer (ITS) region, the external transcribed spacer (ETS) region, and the 5S non-transcribed region of nuclear ribosomal DNA. The phylogeny included 84 of the 86 species currently reported for Mexico and 87% of the total for the genus [10], [15], [16]. Both, fossil and biogeographic data were used to calibrate this phylogeny. Ancestral areas of distribution were reconstructed by using the computer program DIVA [19]. This program optimizes distributions for each node of a phylogeny by favouring vicariance events and minimizing the number of assumed dispersals and extinctions. Its assumptions are well met for this study because *Bursera* species are highly endemic and all of the species have continuous distributions. Centres of origin were identified by restricting the number of unit areas to two in the maxareas option of the optimize command. Once the ancestral centres of origin were reconstructed for each internal node in the phylogeny, we counted the number of diversification events that had taken place in each geographic area. Some species included in the phylogeny such as *B. tomentosa*, *B. nesopola*, and *B. graveolens* were excluded from the analysis because their distribution is mostly outside the specified areas or because their natural distribution is uncertain.

A problem with reconstructing historical geographic distributions based on current distributions is that the older a reconstruction, the less likely it is that the reconstructions are correct. This is because the opportunity for species displacement from the centre of origin increases with time. While this is a possibility for the current investigation, much of *Bursera's* extant diversity originated fairly recently (in the last 15 M years). Thus, reconstructed patterns of spatial diversification should capture an accurate signal of any differential geographic diversification for this speciose genus. Very old species that are likely to have had different distribution in the past such as *B. tecomaca*, whose ancient distribution encompassed Colorado according to fossil information, were omitted from the analysis.

Another complication arises when areas move through time. According to geological evidence, Baja California separated from mainland between 15 and 4 M years ago and the southern cape region is a fragment that became detached from the coasts of Jalisco later attaching to the rest of Baja California [20]–[22]. There are currently 12 *Bursera* species found in the peninsula, including 8 in the cape region. According to the calibrated phylogeny some of these species diverged before the separation of Baja California from mainland, suggesting populations were probably present in Jalisco but subsequently went extinct. Because DIVA does not consider the complexities involved when one area becoming a different one in time, it was assumed that species currently distributed in the tip of Baja were also distributed in Jalisco (southwest region).

Using maps of species distributions we also recorded the number of species currently present in each of the 11 sub-areas as well as the number of species restricted to each sub-area.

## Results

**Figure 3:**
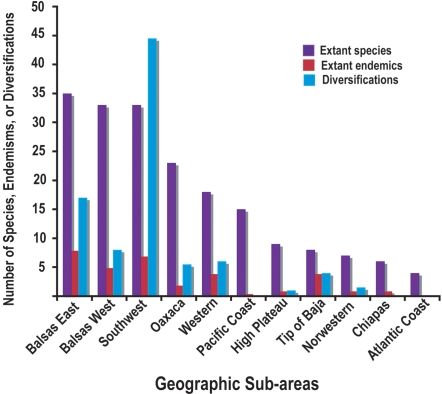
Source and sink areas for diversification. Sub-areas plotted in rank order of number of extant species. The sub-area with the greatest number of extant species and endemisms is different from that with the greatest number of diversifications.

Results show that diversification of *Bursera* seems to have been concentrated in only a few of the 11 geographic sub-areas. Vast areas where species are now present seem to have mostly functioned as diversity sinks (Fig. 3). The majority of the *Bursera* lineages originated in the southwest region, which our results suggest has functioned as a major engine of diversification of the genus. Additional diversification occurred in the two areas that encompass the depression of the Balsas river (Balsas East and Balsas West), and at a lesser extent, in the Oaxaca region. In the past 10 million years about 23 species originated in the southwest sub-area, about 15 in Balsas East, and 8 in Balsas West. In contrast, all of the remaining areas including the Pacific coast, High plateau and Sonoran Desert (Northwest region) have had marginal numbers of diversifications with some of the sub-areas having none.

The spatial pattern of diversification contrasts sharply with the pattern of extant diversity (Fig 3). The sub-area with the greatest number of extant species and endemics is not the one with the greatest number of diversifications. Extant diversity tends to be high everywhere in the southern part of Mexico, particularly in the areas occupied by the Balsas River basin (Balsas East and Balsas West). Endemism is highest in the Balsas East sub-area. Furthermore, several sub-areas such as the Pacific coast, High Plateau, the Northwestern region, and the Chiapas region have a considerable number of extant species (6 to 15) but few (0–2) diversifications.

## Discussion

Our results indicate that centres of diversification for *Bursera* do not coincide well with its current hotspots of species richness or endemism. If areas of the tropical dry forest in the Balsas East region are chosen to be preserved because they contain the highest number of species and endemics, there will be a potential loss of capacity for production of future diversity. The lack of correspondence between the hotspots for diversification and extant diversity is likely to be related to *Bursera*'s history of diversification and geographical expansion, and the topographical heterogeneity of the areas involved.

Previous studies have indicated that *Bursera*'s diversification may be tied to the formation of major mountain systems in Mexico [15], [16]. *Bursera*'s diversification accelerated about 15 million years ago at about the same time as the Western Sierra Madre and the Neovolcanic belt were being formed [23], [24]. These mountains are critical in providing the climatic conditions that maintain tropical dry forests by blocking northern cold fronts. Also, their canyons harbour the prime habitats for the development of tropical dry forest [9]. The genus continues producing a high number of species but it seems that as the main building of these mountains ceased, the geographical expansion of the forest may have come to a halt as well [24]. As these forests began to be saturated with species, *Bursera* started moving into less optimal habitats [15]. For example, recent species such as *B. schlechtendalii*, *B. hindsiana*, *B. morelensis*, and *B. biflora* very likely originated in the dry forest but have invaded drier areas in the High plateau and the Sonoran Desert, which now function as diversity sinks for the genus. The same appears to have happened with other species such as *B. bipinnata* and *B. cuneata*, which now extend into oak forests, or *B. excelsa*, *B. sarcopoda*, *B. arborea*, and *B. heteresthes* that go into sub-humid tropical forests.

The dry forest did not arise all at once, but rather gradually expanded geographically giving the opportunity for older species to colonize newer areas. Diversification of *Bursera* began in the west of Mexico with progressively more eastern diversification following later [25]. This pattern coincides nicely with the proposed history of formation of the Neovolcanic belt. Building of the Neovolcanic axis started about 23 MY ago during the Oligocene and its formation proceeded in several stages, continuing eastward across Mexico until about 2.5 MY ago [23]. Thus, while the initial population of the forest with *Bursera* species in the west involved primarily diversification, the later population of more easterly areas involved both diversification and invasion of extant species from the west. This may partially explain why the Balsas East and Oaxaca regions are high in extant species but lower in diversifications.

Why did most of the diversification occur in the southwest region? The south of Mexico, from the West coast to Oaxaca has good environmental conditions for the persistence of *Bursera* and the dry forest in general. Rains are seasonal, soils have good drainage, and subzero temperatures are infrequent. That is probably the reason why extant *Bursera* diversity is high in these areas [9]. However, the southwest region has an added characteristic that may influence plant diversification. This area is at the intersection of the Western Sierra Madre, which runs from the North, the Neovolcanic axis, which crosses the region in its centre from west to east, and the Southern Sierra Madre, which runs from the south. The result is that the southwest sub-area has a highly interrupted topography with an abundance of deep canyons and heterogeneous environments. A good number of the species start their distribution here, and then trail the Pacific slopes west of the Sierra Madre, while others follow the slopes of the Southern Sierra or start at the Infiernillo region and go through the low canyons and floors of the Balsas basin.

Studies have shown that speciation in *Bursera* is predominantly allopatric and that many species differentiated not only in separate canyons but also at different altitudes in the same canyons [14], [17]. Thus, it is possible that the exceptionally rugged topography of the southwest region has had a positive influence on its rate of diversification and is the reason why it continues producing and exporting a high number of species. Vicariance was probably a result of the building of these canyons and mountains. For example, some authors have speculated that the rising of the Sierra de Taxco that now divides Balsas into the east and west sections was the cause of the divergence between sister species such as *B. lancifolia* and *B. trimera*, and *B. aloexylon* and *B. coyucensis*, whose distribution currently includes only one side of Balsas.

So, how should conservation efforts be focused? Conservation of diversity and endemism hot spots emphasize current diversity [2]. The differences between diversity and diversification mean that this may be transitory in the long run, analogous to protecting species in zoos. While it might sound unusual to try to conserve diversity based on events happened in the past, there may be cases in which the aerographic patterns of diversification have occurred repeatedly for a long time, giving us some kind of assurance that it will continue happening in the same way for at least the near future. In the case of *Bursera*, diversification seems to have been higher in one area for a long time, starting 15 million years ago or perhaps even longer. If not greatly perturbed, there is no reason not to believe that these same patterns of diversification will continue. This approach could be especially useful if there are no other stronger criteria to decide where conservation efforts should be directed. If we had to choose between conserving one of two areas and everything is equal except their history of being sinks or sources of diversification, there would be no harm and perhaps much gain in choosing the source. The long-term maintenance of biodiversity require us preserve its sources, to the extent that these can be accurately determined [8]. This study suggests a way to do so.
